# Na_v_1.7 is the predominant sodium channel in rodent olfactory sensory neurons

**DOI:** 10.1186/1744-8069-7-32

**Published:** 2011-05-10

**Authors:** Hye-Sook Ahn, Joel A Black, Peng Zhao, Lynda Tyrrell, Stephen G Waxman, Sulayman D Dib-Hajj

**Affiliations:** 1 Department of Neurology, Yale University School of Medicine, 333 Cedar Street, New Haven, 06520, USA; 2 Center for Neuroscience and Regeneration Research, Yale University School of Medicine, 333 Cedar Street, New Haven, 06520, USA; 3 Rehabilitation Research Center, Veterans Administration Connecticut Healthcare System, 950 Campbell Avenue, West Haven, Connecticut, 06516, USA

## Abstract

**Background:**

Voltage-gated sodium channel Na_v_1.7 is preferentially expressed in dorsal root ganglion (DRG) and sympathetic neurons within the peripheral nervous system. Homozygous or compound heterozygous loss-of-function mutations in *SCN9A*, the gene which encodes Na_v_1.7, cause congenital insensitivity to pain (CIP) accompanied by anosmia. Global knock-out of Na_v_1.7 in mice is neonatal lethal reportedly from starvation, suggesting anosmia. These findings led us to hypothesize that Na_v_1.7 is the main sodium channel in the peripheral olfactory sensory neurons (OSN, also known as olfactory receptor neurons).

**Methods:**

We used multiplex PCR-restriction enzyme polymorphism, *in situ *hybridization and immunohistochemistry to determine the identity of sodium channels in rodent OSNs.

**Results:**

We show here that Na_v_1.7 is the predominant sodium channel transcript, with low abundance of other sodium channel transcripts, in olfactory epithelium from rat and mouse. Our *in situ *hybridization data show that Na_v_1.7 transcripts are present in rat OSNs. Immunostaining of Na_v_1.7 and Na_v_1.6 channels in rat shows a complementary accumulation pattern with Na_v_1.7 in peripheral presynaptic OSN axons, and Na_v_1.6 primarily in postsynaptic cells and their dendrites in the glomeruli of the olfactory bulb within the central nervous system.

**Conclusions:**

Our data show that Na_v_1.7 is the dominant sodium channel in rat and mouse OSN, and may explain anosmia in Na_v_1.7 null mouse and patients with Na_v_1.7-related CIP.

## Background

Olfactory sensory neurons (OSN; also referred to as olfactory receptor neurons) are bipolar neurons adapted for peripheral odorant signal transduction and transmission centrally to the olfactory bulb. OSN peripheral terminals house a rich array of odorant receptors and a molecular amplification system which boosts receptor potentials produced by short-lived ligand-receptor binding, triggering action potentials that are transmitted centrally along unmyelinated axons which synapse on dendrites of mitral neurons in the well-organized glomeruli in the olfactory bulb within the CNS [1,2]. The voltage-dependent sodium channels that support the initiation and propagation of action potentials in OSN are known to be tetrodotoxin-sensitive (TTX-S) [3]. However, the molecular identity of the TTX-S channels that are expressed in OSN is not known.

Sodium channel Na_v_1.7 has recently emerged as a major target in pain research [4]. This channel is preferentially expressed in peripheral neurons [5-7], and produces a fast-activating and -inactivating, slow-repriming, TTX-S current [8], with slow closed-state inactivation which permits a substantial inward current in response to small, slow depolarizations (ramp current) [9,10]. Na_v_1.7 channels are present in most small unmyelinated fibers within the sciatic nerve [11], and within free nerve endings in the skin [12] close to the predicted peripheral trigger zone. Recently, we have shown that ERK1/2 phosphorylation of the channel hyperpolarizes activation and fast-inactivation of Na_v_1.7 but without changing its current density [13]. The gating properties and subcellular localization suggest that Na_v_1.7 acts as a pre-synaptic threshold channel for firing action potentials which amplifies weak stimuli, for example generator and receptor potentials [14].

Although Na_v_1.7 is being explored as a therapeutic target for pain, recent data support the involvement of Na_v_1.7 in olfactory signaling. Human studies have shown that homozygous or compound heterozygous loss-of-function mutations in *SCN9A*, the gene which encodes Na_v_1.7, cause congenital insensitivity to pain (CIP) [15-17], which is accompanied by anosmia [17-19]. Additionally, global knock-out of Na_v_1.7 in mice is neonatal lethal, reportedly due to lack of feeding [20], consistent with inability of newborn mice to smell mother's milk. We hypothesized that Na_v_1.7 plays a critical role in signal transmission along the olfactory sensory axis from the peripheral olfactory epithelia to the olfactory bulb [4]. We present here molecular and cellular evidence which support the conclusion that Na_v_1.7 is the dominant sodium channel in rodent OSN. Early results of this study have been presented in an abstract form at the 40^th ^annual meeting of the Society for Neuroscience, 2010, program# 848.18.

## Results

### Nav1.7 transcripts are predominant in rat and mouse olfactory epithelium

#### RT-PCR

**Figure 1:**
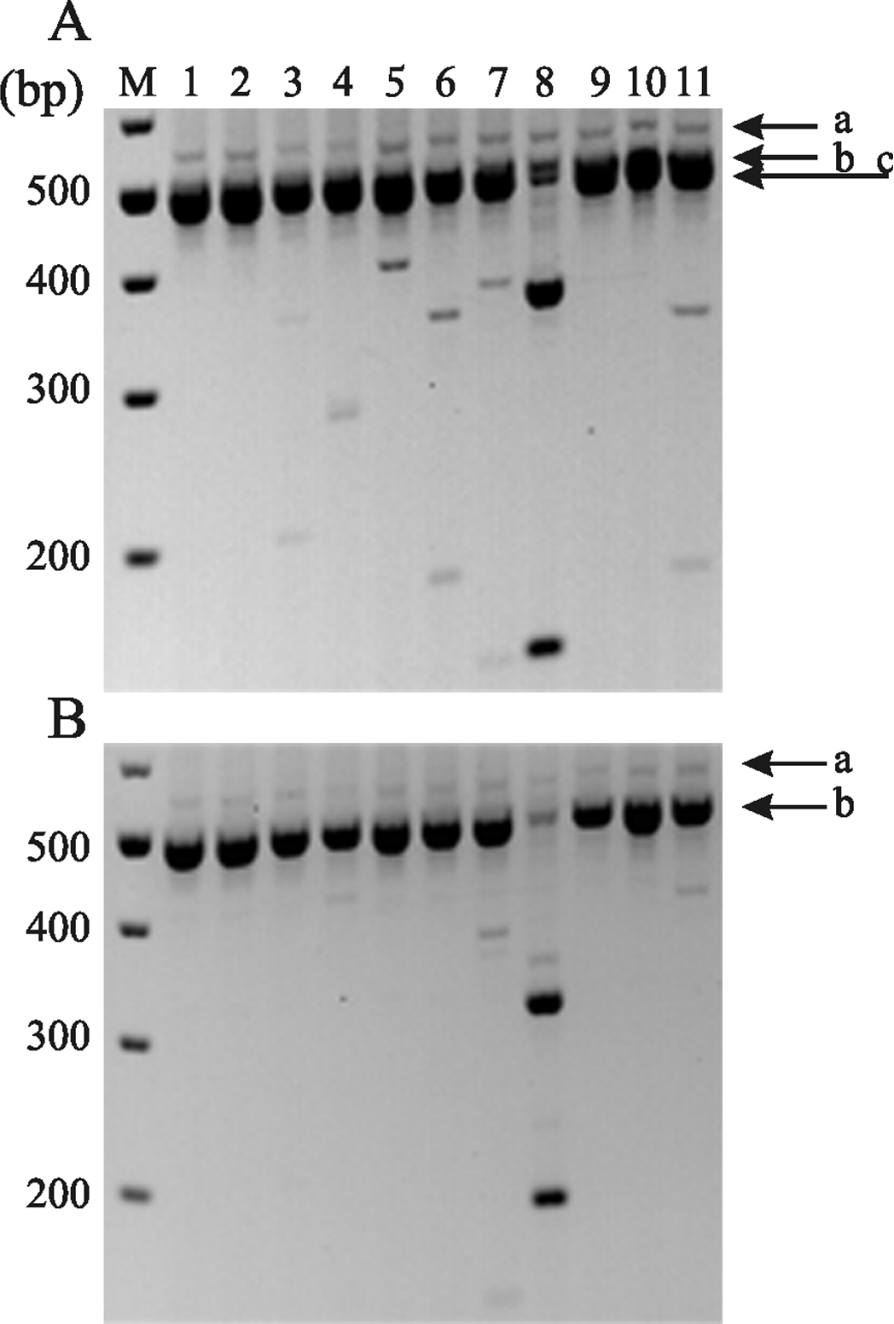
**Restriction analysis of multiplex PCR amplicons of sodium channels from adult rat and mouse olfactory epithelium**. (**A**) Lane M shows 100-bp ladder marker, and lane 1 contains amplicon from rat olfactory epithelium. Bands "a", "b" and "c" (best seen in Lane 8) are consistent with the presence of a potential mixture of sodium channels. Band "a": Na_v_1.1 (558 bp), Na_v_1.2 (561 bp) and Na_v_1.3 (561 bp), band "b": Na_v_1.5 (519 bp); band "c": Na_v_1.6 (507 bp), Na_v_1.7 (501 bp), Na_v_1.8 (480 bp), Na_v_1.9 (468 bp) and Na_x _(501 bp). Lanes 2-11 show results of cutting this DNA with EcoRV (Na_v_1.1), EcoNI (Na_v_1.2), AvaI (Na_v_1.3), NaeI (Na_v_1.4/Na_x_), AccI (Na_v_1.5/1.9), SphI (Na_v_1.6), BamHI (Na_v_1.7/1.8), AflII (Na_v_1.8), EcoRI (Na_v_1.9), and XbaI (Na_x_). (**B**) Lane M shows 100-bp ladder marker, and lane 1 contains amplicons from mouse olfactory epithelium. Bands "a" and "b" are consistent with the presence of a potential mixture of sodium channels. Band "a": Na_v_1.1 (558 bp), Na_v_1.2 (561 bp) and Na_v_1.3 (561 bp); band "b": Na_v_1.6 (510 bp), Na_v_1.7 (501 bp), Na_v_1.8 (480 bp), Na_v_1.9 (471 bp) and Na_x _(501 bp). Lanes 2-11 show the results of cutting this DNA with EcoRV (Na_v_1.1), EcoNI (Na_v_1.2), DraI (Na_v_1.3), PvuI (Na_v_1.4), AgeI (Na_v_1.5), SphI (Na_v_1.6), ScaI (Na_v_1.7), AhdI (Na_v_1.8), EcoRI (, Na_v_1.9) and AlwNI (Na_x_).

Multiplex RT-PCR followed by length polymorphism and restriction enzyme analyses [21-23] were used to investigate the expression of the nine members of Na_v _family of voltage-gated sodium channels [24] in adult rat and mouse olfactory epithelium. Figure 1A (Lane 1) shows amplification products (bands "a", "b" and "c") from rat olfactory epithelium which are consistent with the presence of a potential mixture of Na_v_1.1 (558 bp), Na_v_1.2 (561 bp) and Na_v_1.3 (561 bp) (band a), Na_v_1.5 (519 bp) (band b), Na_v_1.6 (507 bp), Na_v_1.7 (501 bp), Na_v_1.8 (480 bp), Na_v_1.9 (468 bp) and Na_x _(501 bp) (band c). Restriction enzyme analysis of the PCR amplicons (Lanes 2-11) demonstrates that transcripts of Na_v_1.7 are the predominant subtype, with the presence of low levels of transcripts for Na_v_1.2, Na_v_1.3, Na_v_1.5, Na_v_1.6, and the non-voltage-dependent atypical sodium channel Na_x _[25]; transcripts for Na_v_1.1, Na_v_1.4, Na_v_1.8 and Na_v_1.9 are not detected by the restriction enzyme analysis.

Figure 1B (Lane 1) shows amplification products (bands "a", and "b") from mouse olfactory epithelium which are consistent with the presence of a potential mixture of Na_v_1.1 (558 bp), Na_v_1.2 (561 bp) and Na_v_1.3 (561 bp) (band a), Na_v_1.6 (510 bp), Na_v_1.7 (501 bp), Na_v_1.8 (480 bp), Na_v_1.9 (471 bp) and Na_x _(501 bp) (band b); note small difference in length of amplicons for Na_v_1.6 and Na_v_1.9 due to an additional amino acid residue in this region of the mouse channels compared to their rat counterpart. Restriction enzyme analysis of the amplicons demonstrates that transcripts of Na_v_1.7 are the predominant subtype, similar to rat olfactory epithelium (A), with the presence of low levels of transcripts for Na_v_1.3, Na_v_1.6 and Na_x_; transcripts for Na_v_1.1, Na_v_1.2, Na_v_1.4, Na_v_1.5, Na_v_1.8 and Na_v_1.9 are not detected.

We used Na_v_1.7-specific primers to amplify the cDNA of this channel from mouse olfactory epithelial and DRG templates (Table 1). Amplicons were cloned and the identity of the inserts was determined by sequencing. The sequence of the cDNA amplicons confirmed the presence of identical Na_v_1.7 species in the OSN and DRG cDNA templates. Sequencing of clones carrying amplicons which span the two independent alternative splicing events [26], show mutual exclusive splicing of exon 5 isoforms, neonatal (E5N) and adult (E5A), and the alternative 3' splice site selection of exon 11 (E11), leading to the long (E11L) and short (E11S) isoforms, in both DRG and OSN templates. The amino acid sequence of the OSN and DRG Na_v_1.7 cDNAs were identical to previously reported sequences in the GenBank database (accession numbers: BC172147 and BC158048) and match the predicted sequence from the mouse *Scn9a *gene. However, we did not detect the Na_v_1.7 cDNA with alternative splice sites in exons 6 and 9 (accession number: NM_018852) which changes the identity of 14 and 15 amino acids in these exons, respectively.

**Table 1:** Na_v_1.7-specific primers used to amplify cDNA from mouse OSN and DRG templates.

Primer	**Coordinates (accession # **BC172147**)**	Sequence
A Forward	203-221	CTTAGGTAAAGATCCGAAG

A reverse	1374-1353	TGCCAGCAGCACGCAGAGTCTG

B Forward	1267-1289	GCTACACAAGCTTTGACACGTTC

B reverse	2748-2727	GCAGGACTGATAATCCTTCCAC

C Forward	2616-2639	ATGGTACTGAAGTTAATAGCCATG

C Reverse	3744-3722	CTTGGCAGCATGGAAATCTCCGC

D Forward	3613-3636	GTTCTTCAGAGTGCAGCACAGTTG

D reverse	4921-4899	CCAGTGAACAGGATGATGAAGAC

E Forward	4759-4780	GATGCATATTTGACTTAGTGAC

E Reverse	5435-5413	TCCACAGTCCCCTTCCACTGAAC

F Forward	5342-5369	GGATGGACTGCTGGCCCCCATCCTCAAC

F reverse	6264-6243	GTCTTATTAACACGAGTGAGTC

#### *In situ *hybridization

**Figure 2:**
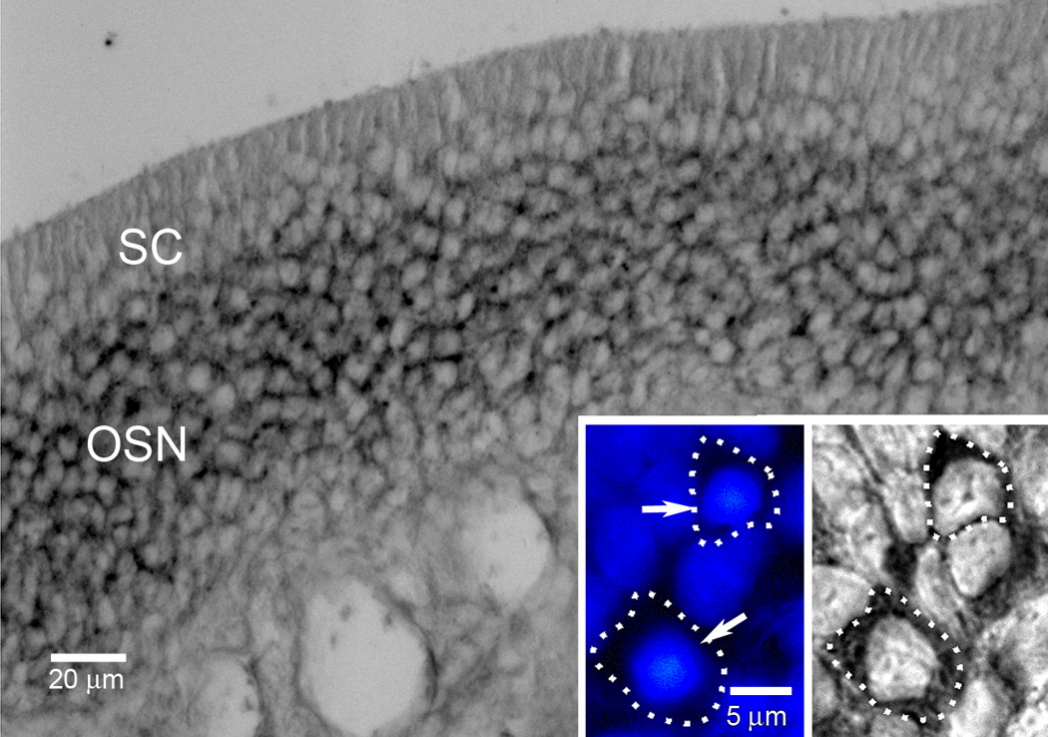
**Na**_**v**_**1.7 mRNA expression in olfactory epithelium using *in situ *hybridization**. *In situ *hybridization signal is exhibited by olfactory sensory neurons (OSN) in the olfactory epithelium. Sustentacular cells (SC) do not express Na_v_1.7 mRNA signal above background levels. Inset: Increased magnification demonstrates Na_v_1.7 in situ hybridization signal in the peri-nuclear cytoplasm of OSN. The cell boundaries of two OSN that exhibit robust Na_v_1.7 signal are demarcated by dotted lines; the DAPI-labeled nuclei of these cells are indicated by arrows.

Since RT-PCR analysis indicated that Na_v_1.7 is the predominant sodium channel isoform within olfactory sensory epithelium, we utilized *in situ *hybridization to determine the cellular distribution of Na_v_1.7 transcripts. As shown in Figure 2, *in situ *hybridization signal was displayed in the region of olfactory epithelium occupied by nuclei of olfactory sensory neurons and not in the area of the nuclei of sustentacular cells. Na_v_1.7 signal was not detected within sub-epithelial regions and Bowman's glands. At higher magnification (Figure 2 inset), *in situ *hybridization signal was localized in juxta-nuclear cytoplasm of olfactory sensory neurons (nuclei labeled with DAPI occupy most of the cellular space).

#### Na_v_1.7 protein in rat olfactory receptor neurons

**Figure 3:**
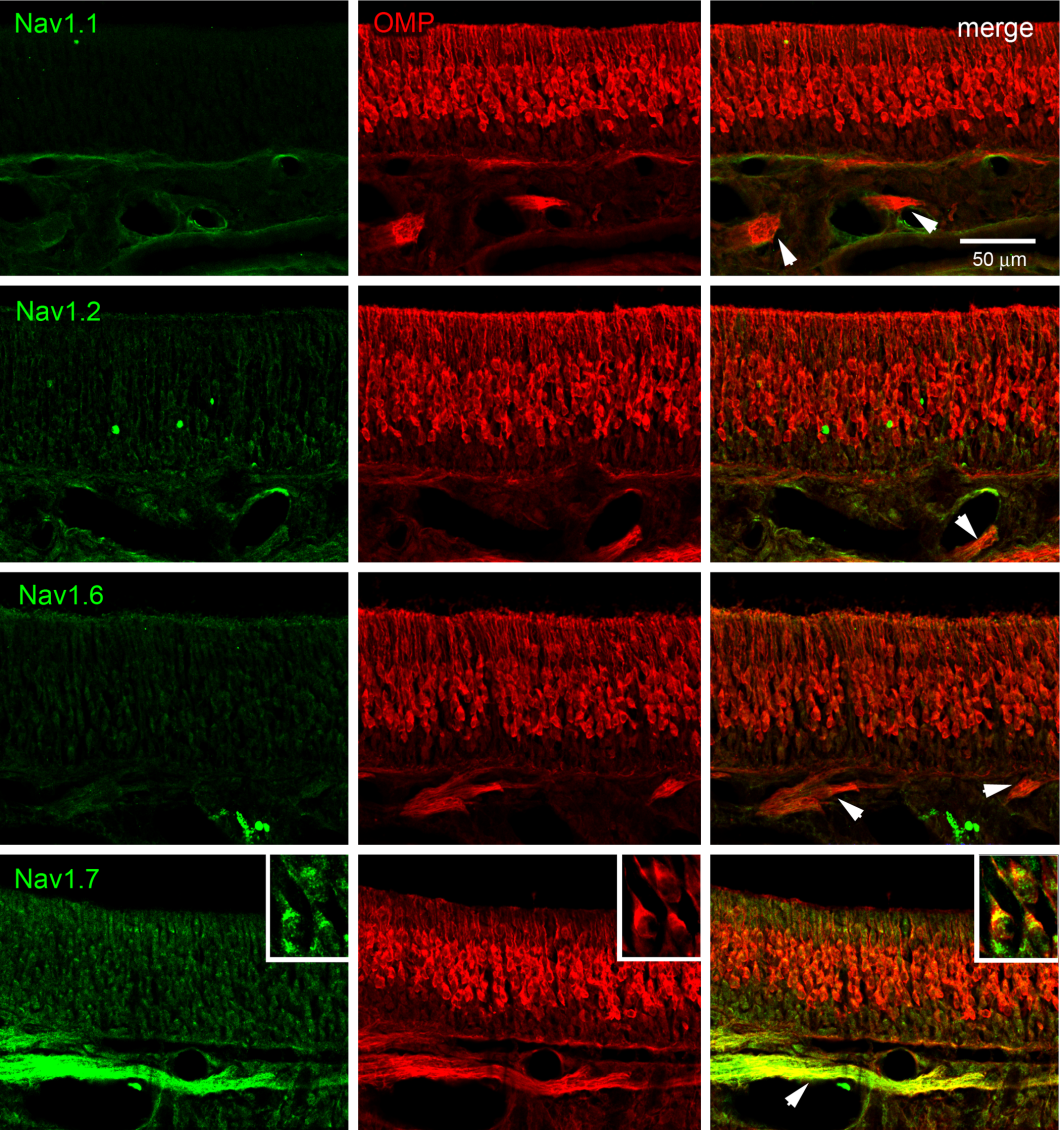
**Sodium channel protein expression in rat olfactory epithelium**. Immunostaining experiments using antibodies specific for sodium channels Na_v_1.1, Na_v_1.2 and Na_v_1.6 show that these channels are not detected within olfactory epithelium or in the subjacent olfactory nerve branches (arrows). In contrast, Na_v_1.7 immunolabeling is displayed in the olfactory epithelium and is robustly expressed within branches of the olfactory nerve (arrows). Insets: At increased magnification, Na_v_1.7 immunoreactivity (green) is displayed by OMP-positive OSN.

We examined the distribution within olfactory epithelium of sodium channel proteins Na_v_1.1, Na_v_1.2, Na_v_1.6 and Na_v_1.7 channels which have been detected by the RT-PCR assay and for which robust isoform-specific antibodies are available. As shown in Figure 3, sodium channels Na_v_1.1, Na_v_1.2 and Na_v_1.6 were not detected within OSN or the subjacent nerve within the olfactory epithelium (Figure 3). In contrast, Na_v_1.7 signal was detected within OSN and branches of the olfactory nerve exhibited robust Na_v_1.7 immunolabeling (Figure 3). At higher magnification (Figure 3 insets), Na_v_1.7 immunoreactivity is clearly present within mature OSN which express olfactory mature protein (OMP^+^) in the olfactory epithelium

#### Na_v_1.7 protein in rat olfactory bulb

**Figure 4:**
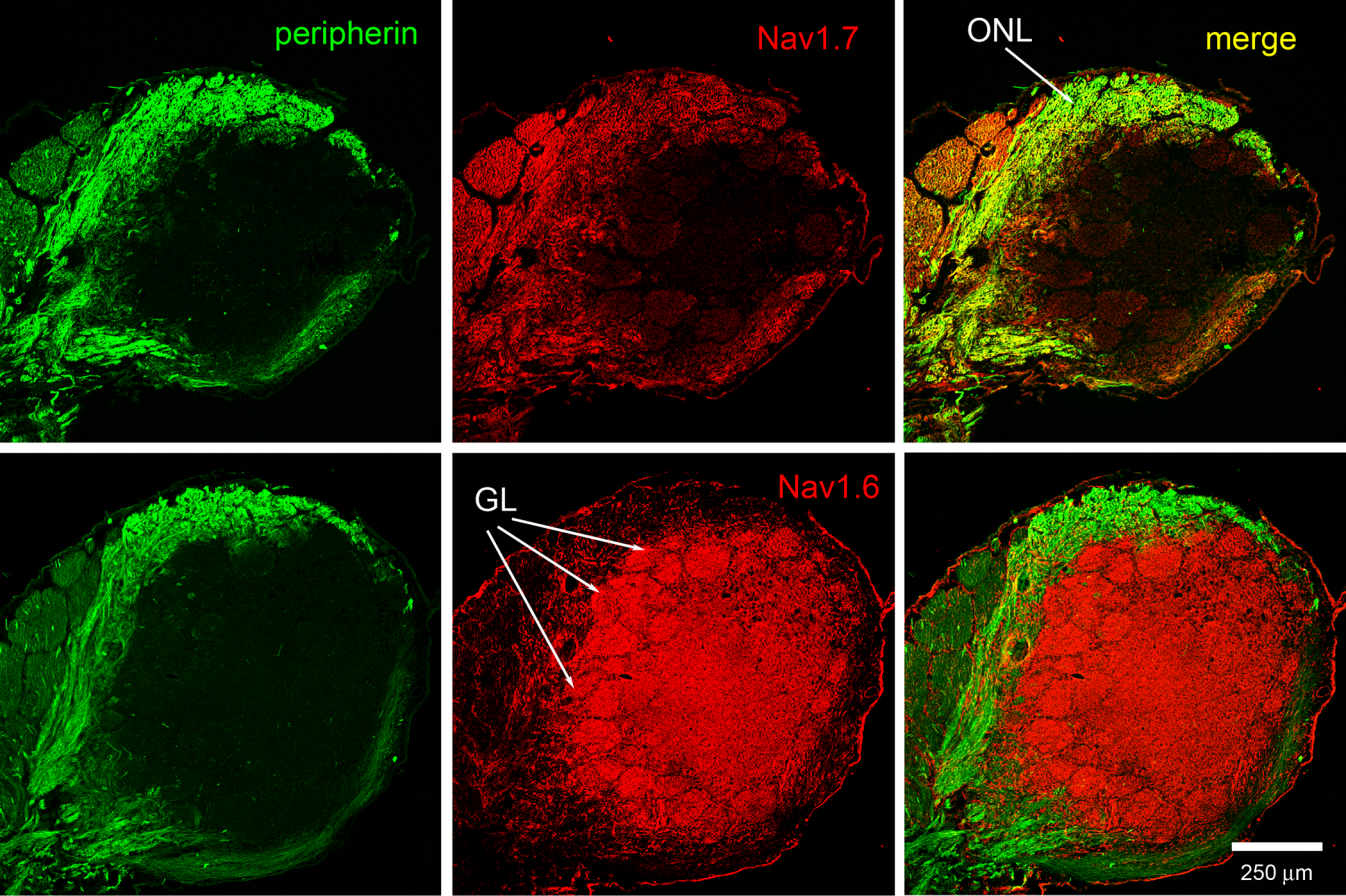
**Sodium channels Na**_**v**_**1.6 and Na**_**v**_**1.7 expression in olfactory nerve layer of rat olfactory bulb**. Immunolabeling experiments show that sodium channel Na_v_1.7 (red) is co-localized (yellow) with peripherin (green) in fibers of the olfactory nerve layer (ONL). In contrast, only limited Na_v_1.6 immunoreactivity is displayed within the olfactory nerve layer, with robust labeling of the glomeuli (GL).

The slender (0.1-0.3 mm diameter) axons of OSN traverse from the olfactory epithelium to the surface of the olfactory bulb (olfactory nerve layer) and then penetrate the bulb to synapse with dendrites of mitral cells within glomeruli (see [27]). Sections of olfactory bulb reacted with antibodies specific to Na_v_1.7 and peripherin, a marker of unmyelinated fibers [28], exhibit robust co-localization of Na_v_1.7 and peripherin within the olfactory nerve layer (Figure 4). Notably, Na_v_1.7 immunolabeling is not detected within the mitral cell layer of the olfactory bulb. In contrast to the labeling pattern of Na_v_1.7, olfactory bulb sections probed with Na_v_1.6 antibodies exhibit a general paucity of Na_v_1.6 labeling within the olfactory nerve layer, while there is robust Na_v_1.6 immunoreactivity within the glomerular layer of the olfactory bulb (Figure 4).

**Figure 5:**
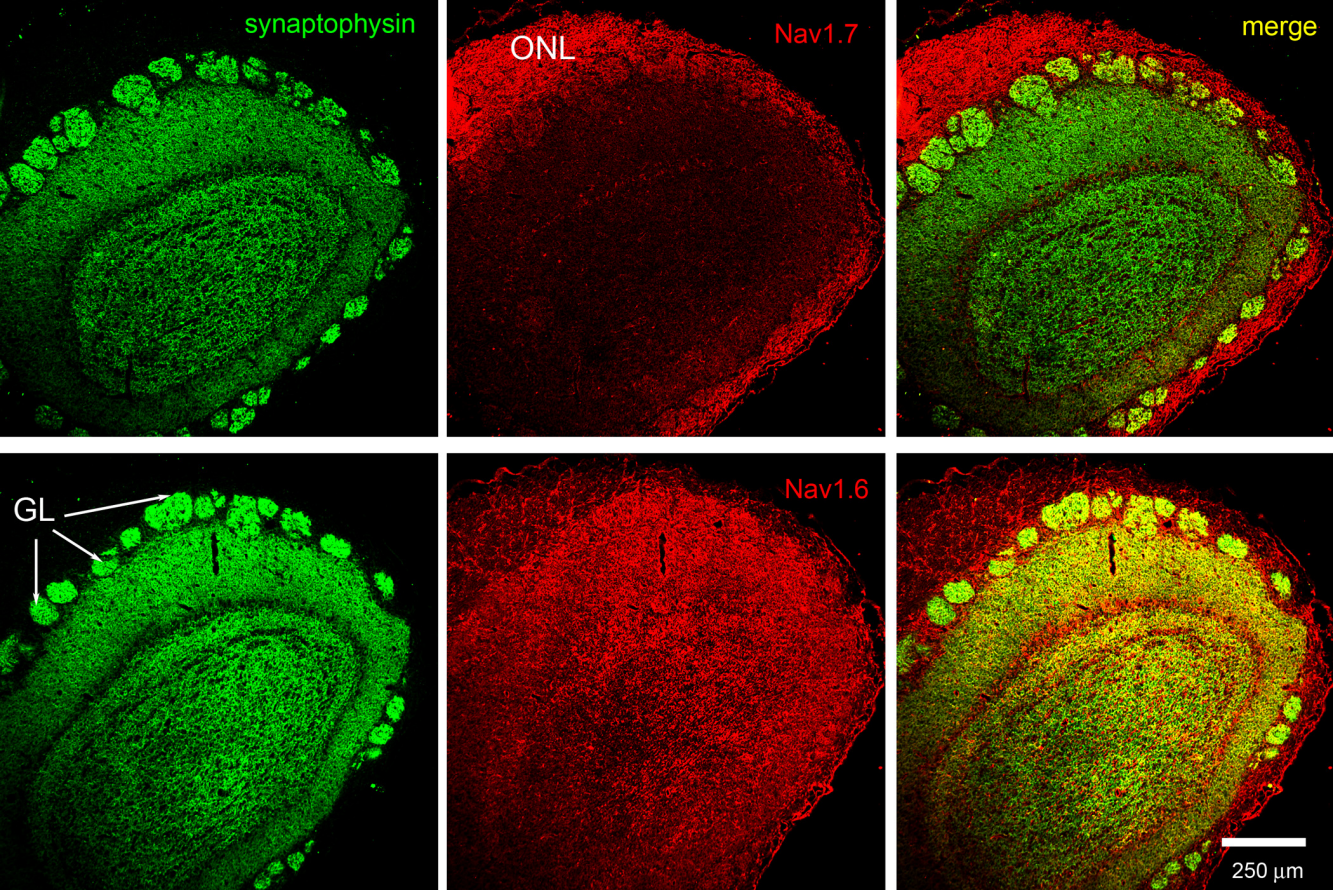
**Sodium channels Na**_**v**_**1.6 and Na**_**v**_**1.7 expression in rat glomerular layer of olfactory bulb**. Immunolabeling experiments show that the synaptophysin-positive (green) glomeruli of the olfactory bulb exhibit extremely limited Na_v_1.7 (red) immunoreactivity. In contrast, Na_v_1.6 (red) exhibits robust immunoreactivity within the synptophysin-positive (green) glomeruli of the olfactory bulb.

**Figure 6:**
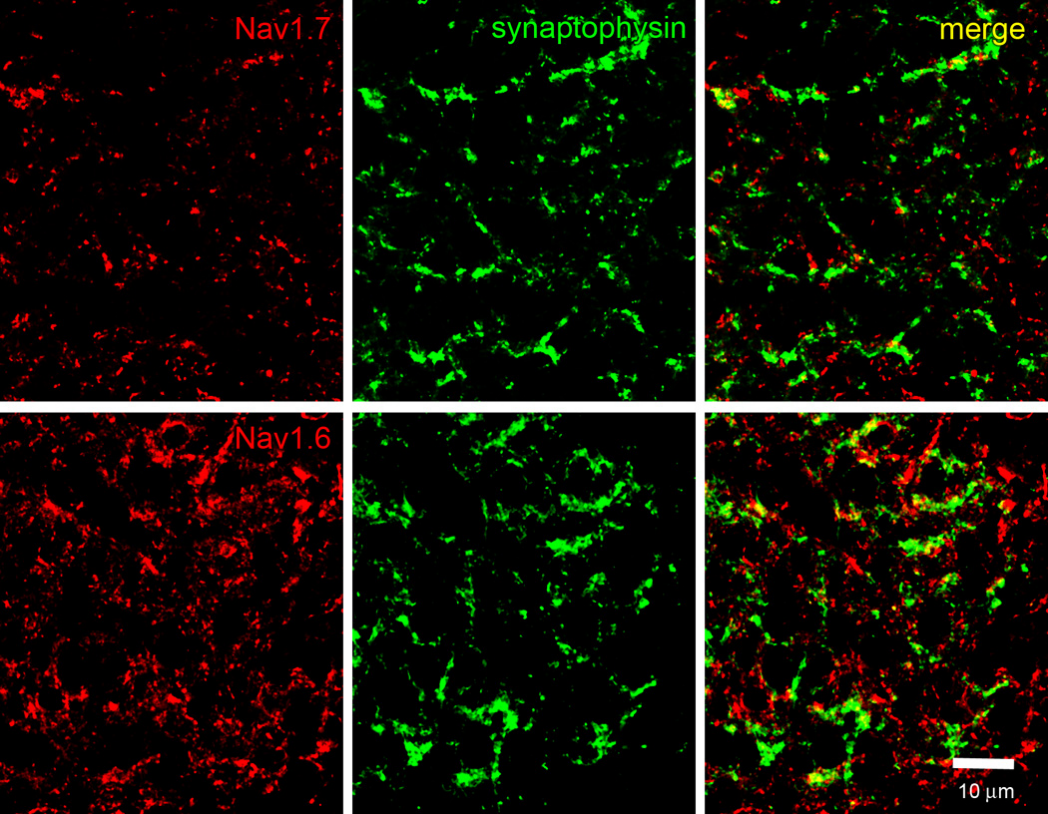
**Sodium channels Na**_**v**_**1.6 and Na**_**v**_**1.7 expression in terminal boutons of rat glomerular layer of olfactory bulb**. Na_v_1.7 (red) immunoreactivity within the olfactory bulb glomerulus is punctate and extremely limited, and only occasional co-localization of Na_v_1.7 with synaptophysin-positive (green) terminal boutons is observed. In contrast, there is robust Na_v_1.6 (red) immunoreactivity within the glomerulus, although there is limited co-localization with synaptophysin-positive (green) terminal boutons within the glomerulus of the olfactory bulb.

The differential and complementary pattern of Na_v_1.7 versus Na_v_1.6 immunolabeling in the olfactory bulb is readily apparent in sections reacted with sodium channel antibodies and the synaptic marker synaptophysin (Figure 5). Robust Na_v_1.7 immunolabeling is displayed within the olfactory nerve layer, but limited Na_v_1.7 immunoreactivity is detected within glomeruli. In contrast, the expression of Na_v_1.6 is nearly the inverse of that displayed by Na_v_1.7, with a paucity of Na_v_1.6 immunolabeling within axons of the olfactory nerve layer and robust immunoreactivity within glomeruli (Figure 5). Imaging the glomeruli at higher magnification (Figure 6), shows that, within the glomeruli, Na_v_1.7 is present in discrete punctate structures around 1 μm in diameter suggesting the presence in OSN axons. The lack of co-localization with synaptophysin in the glomeruli suggests that the density of Na_v_1.7 within synaptic boutons is below levels of detection or that this channel is totally absent from these boutons. In contrast, Na_v_1.6 is present in larger foci, occasionally overlapping with synaptophysin but more commonly not overlapping, consistent with the presence of Na_v_1.6 in post-synaptic dendrites and/or astrocytic processes where Na_v_1.6 has been detected [29].

**Figure 7:**
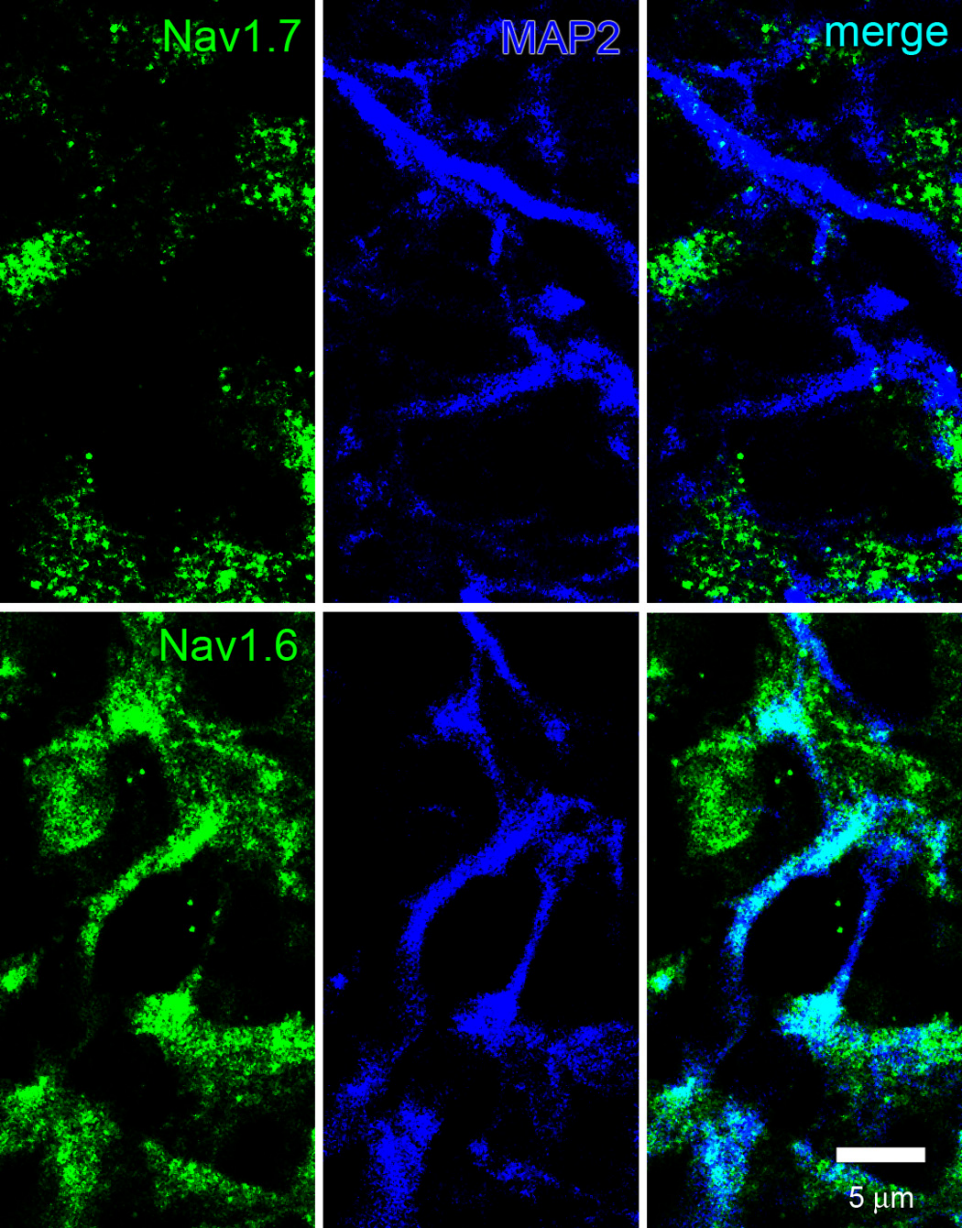
**Sodium channel expression within glomerulus dendrites in rat olfactory bulb**. MAP2-positive (blue) dendrites do not exhibit detectable Na_v_1.7 (green) immunoreactivity. In contrast, Na_v_1.6 immunolabeling is displayed within most MAP2-positive dendrites (co-localization is cyan).

The complementary distribution of Na_v_1.7 in OSN and mitral neurons within the glomeruli is supported by co-localization studies with MAP2, a marker of dendrites. Figure 7 shows a lack of co-localization of Na_v_1.7 and MAP2, consistent with the restriction of this channel to pre-synaptic OSN structures. In contrast, Figure 7 shows a significant co-localization of Na_v_1.6 and MAP2, consistent with its expression in mitral and other post-synaptic neurons in the olfactory bulb.

#### Activation and steady-state fast-inactivation of sodium currents in mouse OSNs

**Figure 8:**
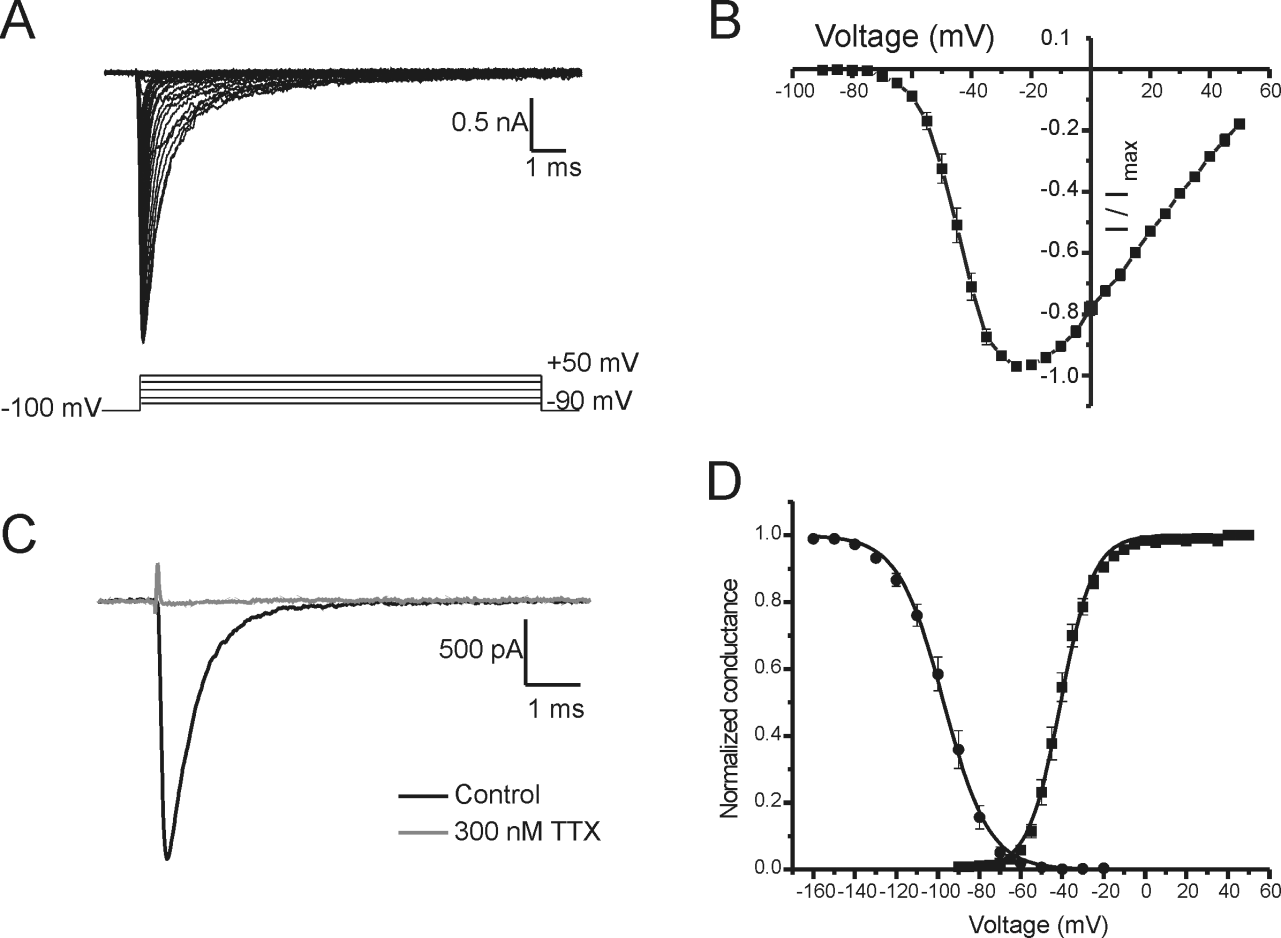
**Voltage-dependent activation and fast-inactivation for sodium currents in mouse OSN**. (**A**), sodium currents were evoked by 100 ms depolarizing pulses from -90 mV to +50 mV in steps of 5 mV every 5 s from a holding potential of -100 mV. (**B**), Normalized peak current-voltage relationship. (**C**), The inward current was completely blocked by 300 nM TTX. (**D**), Voltage dependence of activation and steady-state fast-inactivation. To measure steady-state fast-inactivation, sodium currents were elicited by 500 ms prepulses from -160 mV to -20 mV stepped by 10 mV and followed by a 40 ms depolarizing pulse to -20 mV from a holding potential of -100 mV. The activation and fast-inactivation curves were obtained from a Boltzmann fit to the normalized conductance.

Rat OSNs have been reported to express TTX-sensitive sodium currents [3]. We recorded inward sodium currents in adult mouse OSNs (8-13 μm diameter) using the whole-cell voltage-clamp method (Figure 8). To characterize the activation kinetics, sodium currents in acutely isolated OSNs (average peak amplitude of 1.7 ± 0. 2 nA, *n *= 17) were elicited by 100 ms depolarizing pulses from -90 mV to +50 mV in 5 mV increments from a holding potential of -100 mV (Figure 8A). As shown in the normalized current-voltage relationship (Figure 8B), sodium currents in OSN were activated at potentials positive to -70 mV and reached a peak near -25 mV, with a reversal potential of 63.1 ± 1.1 mV (*n *= 17). The rapidly inactivating inward sodium current was evoked by a step voltage to -20 mV from a holding potential of -100 mV and was completely blocked by 300 nM TTX (Figure 8C). The voltage midpoint (V_1/2 _= -40.9 ± 1.2 mV, n = 17) and slope factor (*k *= 7.1 ± 0.5 mV) of activation were obtained from a Boltzmann fit of normalized conductance (Figure 8D). The activation voltage-dependence for sodium currents in OSNs (V_1/2 _= - 40.9 mV) is more hyperpolarized than that (V_1/2 _range: -16 to -29 mV) for Na_v_1.7 currents in HEK293 cells [9,30-40].

To examine the voltage-dependence of steady-state fast-inactivation, OSNs were held at -100 mV and the sodium currents were induced by a double pulse protocol of 500 ms prepulses from -160 mV to -20 mV in 10 mV increments, followed by a 40 ms depolarizing pulse to -20 mV to measure the fraction of available channels. The fast-inactivation curve was obtained from a Boltzmann fit to the normalized current (Figure 8D). The voltage midpoint (V_1/2_) and slope factor (*k*) of steady-state fast-inactivation were -96.4 ± 2.1 mV and 8.9 ± 0.5 mV (*n *= 17). Similar to activation properties, the steady-state fast-inactivation for sodium currents in OSNs is also more hyperpolarized than that (V_1/2 _range: -71 to -83 mV) for Na_v_1.7 currents in HEK293 cells [9,30-40].

## Discussion

We show here that Na_v_1.7 is the predominant transcript in adult rat and mouse olfactory epithelium, with low abundance of Na_v_1.6 transcripts. Immunostaining of olfactory epithelium and the olfactory nerve show that Na_v_1.7 is the main sodium channel which accumulates in the thin unmyelinated fibers, with undetectable Nav1.6 immunolabeling. Co-immunostaining with the synaptic marker synaptophysin reveals a complementary distribution of Na_v_1.7 and Na_v_1.6 channels, with accumulation of Na_v_1.7 in presynaptic axons, and Na_v_1.6 in processes of mitral and granule neurons within glomeruli of the olfactory bulb. The limited Na_v_1.7 immunoreactivity at the presynaptic axon termini within the glomeruli may possibly reflect the dispersion of axons and their small diameter, and the robust Nav1.6 immunostaining within the glomeruli reflects the abundant expression of this channel within processes of mitral and granule neurons. Weiss et al. [41] recently reported the presence of Na_v_1.7 within mouse and human OSN, and observed Na_v_1.7 immunoreactivity that extended from the cell bodies of mouse OSN to their axons within olfactory glomeruli. Their results, like ours, indicate that, while Na_v_1.7 is the major sodium channel within OSN, it is not detectable in the mitral and granule neurons that receive synaptic inputs from the OSN. Consistent with this conclusion, they report that there is no synaptic transmission of the electric impulse from OSN to the post-synaptic neurons in mice where Na_v_1.7 is knocked-out in mature OSN that express the olfactory marker protein. In the aggregate, these data support the conclusion that Na_v_1.7 is the predominant sodium channel responsible for peripheral odorant signaling to the olfactory bulb.

Transient inward currents in rat and mouse OSNs are completely blocked by 100 nM TTX or by substitution of choline for external sodium, and action potentials are blocked by 100 nM TTX ([3,41,42] and this study), indicating that OSN excitability is dependent upon a TTX-S sodium channel. Consistent with the electrophysiological data, our molecular and immunostaining data show that Na_v_1.7, a TTX-S channel, is the predominant sodium channel in OSNs and their unmyelinated axons. Low levels of other TTX-S sodium transcripts could be amplified from olfactory epithelial cDNA templates (Figure 1A and 1B), but weak or no immunostaining for these channels was detectable in OSN and in axons within the olfactory nerve (Figure 3). While the cellular origin of sodium channel transcripts other than Na_v_1.7 is difficult to identify, one possibility is that low expression levels of these channels occurs in OSN, but the level of the channel protein is below the detection of our immunostaining assays. Alternatively, other cell types within the olfactory epithelium may express sodium channels other than Na_v_1.7. Irrespective of the source of these weakly-expressed channels, the vast abundance of Na_v_1.7 in the OSN points to a critical role of this channel in olfactory signal transmission, and that the presence of other sodium channels does not appear to be sufficient to rescue olfaction in humans and mice which lack Na_v_1.7.

The ability of Na_v_1.7 to boost subthreshold stimuli, for example odorant-induced receptor potential depolarization of OSN membrane, is consistent with its role as a threshold channel for firing action potentials in neurons [14]. Single openings of the metabotropic odorant receptors have been shown to be sufficient to generate action potentials [43], although a recent study [44] has estimated that a single odorant binding event results in ~0.034 pA current while the threshold for action potential is ~1.2 pA. An elaborate Ca^2+^- and Cl^-^-based signaling amplification system in the cilia has been reported to boost the odorant receptor potential for successful initiation of action potentials [1,2]. However, the abundant expression of Na_v_1.7 with its demonstrable ability to boost weak depolarizations, and weak expression of other sodium channels in rodent and human OSN ([41] and this study), supports the conclusion that Na_v_1.7 plays a central role in action potential transmission along the peripheral olfactory nerve axis.

While the molecular and immunostaining data show dominant expression of Na_v_1.7 channels in rodent and human OSN ([41] and this study), whole-cell patch-clamp recordings of mouse OSN show a TTX-S current with hyperpolarized activation and inactivation; threshold for activation was near -70 mV with a peak around -25 mV and V_1/2 _of -41 mV (Figure 8). Data presented in Figure 3B by Weiss et al [41] are in agreement with hyperpolarized voltage dependence for activation (threshold and peak) in OSN. These data are consistent with a published report of V_1/2 _of -48 mV from isolated P5-P15 rat OSN [45]. The voltage-dependence of activation for sodium current in OSNs is more hyperpolarized than those for human Na_v_1.7 currents (V_1/2 _range: -16 to -29 mV) in HEK293 cells [9,30-40] or DRG neurons [10,46], or of TTX-S currents in native DRG neurons (-23 to -28 mV) [47-49]. Similarly, a wide range of V_1/2 _for steady-state inactivation of sodium current has been reported for rat OSNs: -96 mV for adult mouse (this study), -87 mV for P5-P15 rat [45], and -110 mV [50], -107 mV [51], and -105 mV [52] for adult rat OSNs. The wide range of reported V_1/2 _may arise from the use of neurons from neonatal, juvenile or adult rats, different recording buffers, time in culture and other technical issues. However, the V_1/2 _of -96 mV that we obtained is hyperpolarized compared to those (V_1/2 _range: -71 to -83 mV) reported for Na_v_1.7 current in HEK cells [9,30-40] or DRG neurons [10,46,53] or the TTX-S currents (-66 to -72 mV) in native DRG [47-49]. Since we report in this study that the sequence of the Na_v_1.7 cDNA is identical in mouse OSN and DRG templates, modulation of the Na_v_1.7 channels by post-translational modification and possible interaction with cell-specific channel partners, rather than a different Na_v_1.7 splicing isoform, is likely responsible for the altered gating properties of this channel in OSN versus DRG neuronal backgrounds.

Using RT-PCR, we also observed (Figure 1A) Na_v_1.6 mRNA at low levels in mouse and rat olfactory epithelium (the only detectable sodium channel transcript other than Na_v_1.7 in mouse tissue), but found no detectable immunostaining signal in rat OSN or olfactory nerve axons, which suggest a limited contribution of this channel to peripheral olfactory signal transmission. Studies on *Scn8a*^*medtg *^mice which lack Na_v_1.6 channels support our view that Na_v_1.7 is essential for olfaction in mice. While global knock-out of Na_v_1.7 in mice is neonatal lethal [20], total loss of Na_v_1.6 in mice is juvenile lethal although these mice are indistinguishable from WT or heterozygote littermates in terms of feeding and open field behavior for the first 10-14 days after birth [54-56]. Neonatal lethality of Na_v_1.7 knock-out mouse has been linked to lack of feeding [20], while death of Na_v_1.6 knock-out mouse is linked to muscle degeneration [54-56]. These data are consistent with a minor role for Na_v_1.6 in OSN excitability and olfactory signal transmission, at least within the first two weeks after birth.

Recent data have shown that Na_v_1.7, which is normally considered a threshold sodium channel [14], is critical to nerve signal transduction and transmission in two sensory neuronal pathways: nociception and olfaction. The predominant expression of Na_v_1.7 in OSN ([41] and this study), compared to other channels, provides a reasonable explanation for anosmia in human subjects [15,17-19] and mice [20] when this channel is not functional. Thus, Na_v_1.7 appears to be critically important for olfactory signaling by OSN. In contrast to OSN where Na_v_1.7 expression predominates, Na_v_1.7 is co-expressed with several other channels within DRG neurons which signal pain [4], and these channels are distributed to the peripheral free endings of the axons in the epidermis [12]. These observations, together with the profound loss of pain sensibility in CIP, point to a dominant role of Na_v_1.7 in pain-signaling, although the exact mechanism is not well understood. Intriguingly, Na_v_1.7 is present in sympathetic neurons and gain-of-function mutations that depolarize resting membrane potential cause hypoexcitability of these neurons [57]. However, Na_v_1.7-related CIP patients do not report significant sympathetic dysfunction [15-18], thus it appears that Na_v_1.7 does not play an equally central role in signal transduction/transmission in sympathetic neurons. These data show that the contribution of Na_v_1.7 channels to neuronal activity appears to be neuronal-type dependent.

## Conclusions

We present here molecular and immunolabeling data that demonstrate that Na_v_1.7 is the predominant sodium channel in OSN and along olfactory nerve fibers. Gain-of-function mutations of Na_v_1.7 cause hyperexcitability of DRG neurons, underlying pain symptoms in inherited erythromelalgia and PEPD; however, it has not been reported that patients with these disorders also manifest hyperosmia. In contrast, patients with Na_v_1.7-related CIP report anosmia, and the data presented in this study provide a molecular basis for anosmia in these patients. Na_v_1.7-specific blockers are being pursued as a highly targeted approach for the treatment of pain. Our data suggest that hyposmia or anosmia are potential side effects that need to be taken into consideration in the clinical application of these therapeutics.

## Materials and methods

### Animal care

Sprague-Dawley male rats (adult, 225-250 gm, Harlan, Indianapolis, IN) and C57BL/6 mice (adult, 25-30 gm, Harlan) were housed under a 12 hr light/dark cycle in a pathogen-free area with *ad libitum *access to water and food. The experimental procedures were approved by the VA Connecticut Healthcare System Institutional Animal Care and Use Committee, in accordance with NIH guidelines and conform to the guidelines of the Committee for Research and Ethical Issues of the IASP.

### RNA extraction and cDNA synthesis

Rats and mice were deeply anaesthetized with CO_2_, decapitated, and olfactory epithelium was quickly removed and immediately frozen in liquid nitrogen. Total RNA was extracted using RNeasy mini kit (Qiagen, Valancia, CA) and RNA was eluted in 30-50 μl of H_2_O. First strand cDNA was reverse transcribed in a 20 μl reaction volume including 7 μl total RNA, 200 ng random primers and 200 U SuperScript III reverse transcriptase (Invitrogen, Carlsbad, CA), and 40 U RNase inhibitor (Roche Biosciences, Indianapolis, IN). The buffer consisted of: 50 mM Tris-HCl (pH 8.3), 75 mM KCl, 3 mM MgCl_2_, 5 mM DTT and 0.5 mM dNTP. The reaction proceeded at 25°C for 5 min, 50°C for 90 min and then terminated by heating to 70°C for 15 min. A parallel reaction was performed as a negative control by substituting sterile water for the reverse transcriptase enzyme (data not shown).

### Multiplex PCR and Restriction endonuclease analysis

A multiplex PCR was used to amplify Na_v _channel transcripts (Na_v_1.1, Na_v_1.2, Na_v_1.3, Na_v_1.4, Na_v_1.5, Na_v_1.6, Na_v_1.7, Na_v_1.8, Na_v_1.9, and Na_x_) which may be present in the cDNA pool as previously described [21-23]. Primers were designed against highly conserved sequences in domain I of sodium channel α subunits. Sequences of the four forward and three reverse primers (F1-F4 and R1-R3) are as follows: F1 5'-AATCCCTGGAATTGGTTGGA-3', F2 5'-GACCCRTGGAACTGGCTGGA-3', F3 5'-GACCCGTGGAACTGGTTAGA-3', F4 5'-GATCTTTGGAACTGGCTTGA-3'; R1 5'-CAAGAAGGCCCAGCTGAAGGTGTC-3', R2 5'-GAGGAATGCCCACGCAAAGGAATC-3', R3 5'-AAGAAGGGACCAGCCAAAGTTGTC-3'. Amplification was performed in a 60 μl reaction volume using 4 μl first-strand cDNA, 1-3 μM of each primer and 5 U of Expand Long Template DNA polymerase enzyme mixtures (Roche). The PCR reaction buffer contained 2.75 mM MgCl_2 _and detergents. Amplification was carried out in two stages using a programmable thermal cycler (PTC-200, MJ Research, Cambridge, MA). First, a denaturation step at 94°C for 2 min, an annealing step at 57°C for 2 min and an elongation step at 68°C for 2 min. Second, a denaturation step at 94°C for 30 sec, an annealing step at 57°C for 45 sec and an elongation step at 68°C for 45 sec. The second stage was repeated 39 times for a total of 40 cycles, with the elongation step in the last cycle extended to 10 min. Control PCR reactions in which the template was substituted by H_2_O produced no amplification products (data not shown).

The identity of the α-subunits expressed in rat or mouse olfactory epithelium were determined by a combination of length polymorphism and restriction endonuclease analysis of the PCR products. Typically 1/20th of the PCR products were digested for 1 hr at the recommended temperature and the products resolved by electrophoresis in a 2% agarose gel. Fragment sizes were determined by comparison to a standard 100-bp ladder molecular weight marker (Invitrogen). DNA was visualized by ethidium bromide fluorescence and the gel image was digitized by a Kodak Image Station 440 CF (Kodak, Rochester, NY).

The Na_v_1.7 cDNA from mouse OSN and DRG templates were amplified using 6 primer pairs which amplified overlapping fragments (Table 1). Amplicons were purified using spin columns (Qiagen), and cloned into pGEM-Teasy vectors (Promega Inc.). The identity of the fragments was determined by sequencing of both strands at the W. Keck core facility of Yale School of Medicine. Sequence analysis was done using Lasergene and BLAST software.

### *In situ *hybridization

Rats were deeply anesthetized with ketamine/xylazine (80/5 mg/kg, i.p.) and transcardially perfused with PBS and then ice-cold fixative solution containing 4% paraformaldehyde in 0.14 M Sorensen's phosphate buffer, pH 7.4. Olfactory epithelium was removed and fixed for an additional 2-4 hr in the fixative solution and then transferred to a 4% paraformaldehyde solution containing 30% sucrose overnight at 4°C. Twelve micron cryosections were cut and tissue processed for non-radioactive *in situ *hybridization detection of Na_v_1.7 mRNA as previously described [58]. Briefly, sections were deproteinized with proteinase K (10 μg/ml), acetylated with 0.25% acetic anhydride in 0.1 M triethanolamine, and incubated in pre-hybridization buffer (50% formamide, 5 × SSC, 5 × Denhardt's solution, 100 μg/ml salmon sperm DNA; Sigma, St. Louis, MO) for 1 hr at room temperature followed by hybridization buffer (50% formamide, 10% dextran sulfate, 5 × SSC, 1 × Denhardt's solution, 100 μg/ml salmon sperm DNA, Sigma) containing digoxigenin (DIG)-UTP-labeled Na_v_1.7 (1.0 ng/μl) riboprobes overnight at 58°C. The slides were then sequentially incubated in: (1) 4 × SSC, 5 min; (2) 2 × SSC, 2 × 10 min each; (3) RNase A solution (20 μg/ml; Sigma) in 10 mM Tris/500 mM NaCl/1 mM EDTA, pH 8.0, for 45 min at 37°C; (4) 2 × SSC, 2 × 10 min each; (5) 0.2 × SSC, 3 × 20 min each at 58°C; (6) 100 mM Tris/150 mM NaCl, pH 7.5, 1 min; (7) blocking solution, containing 100 mM Tris/150 mM NaCl/2% normal sheep serum/1% BSA, 30 min, (8) alkaline phosphatase-labeled anti-DIG antibody (1:500 in blocking solution; Roche) overnight at 4°C; (9) 100 mM Tris/150 mM NaCl, pH 7.5, 4 × 5 min each; (10) 100 mM Tris/100 mM NaCl/50 mM MgCl_2_, pH 9.5, 4 × 5 min each; (11) NBT/X-phos solution [384 μg/ml *ρ*-nitro-blue tetrazolium chloride (NBT) and 188 μg/ml 5-bromo-4-chloro-3-indolyl phosphate (X-phos) in 100 mM Tris/100 mM NaCl/50 mM MgCl_2_, pH 9.5]. The reaction was stopped by rinsing in 10 mM Tris/1 mM EDTA, pH 8.0. Sections were incubated with 300 nM 4', 6-diamidino-2-phenylindole (DAPI) to label olfactory sensory nuclei.

### Immunocytochemistry

Rats were deeply anesthetized with ketamine/xylazine (80/5 mg/kg, i.p.) and transcardially perfused with 0.01 M PBS (pH 7.4) followed by ice-cold 4% paraformaldehyde in 0.14 M Sorensen's phosphate buffer (pH 7.4). The olfactory epithelium was removed, immersion-fixed for an additional 20 min (total fixation time 30 min) and cryoprotected with 30% (w/v) sucrose in PBS overnight at 4°C. Ten-μm thick cryosections were mounted on slides (Fisher, Pittsburgh, PA) and processed for detection of Na_v_1.7 protein as described previously [59]. In brief, sections were incubated in the following (1) blocking solution (PBS containing 5% cold water fish skin gelatin, 3% normal donkey serum, 2% BSA, 0.1% Triton X-100, and 0.02% sodium azide) for 15 min at room temperature; (2) primary antibody(ies) singly or in combination [mouse anti-Na_v_1.1 (1:100, Antibodies, Inc., Davis, CA); mouse anti-Na_v_1.2 (1:100, Antibodies, Inc.); rabbit anti-Na_v_1.6 (1:100, Sigma); rabbit anti-Na_v_1.7 (1:250, Y083 [60]); goat anti-olfactory mature protein (OMP) (1:200 Wako, Richmond, VA); mouse anti-peripherin (1:1000, Abcam), and mouse anti-synaptophysin (1:50, GeneTex, Irvine, CA)] in blocking solution overnight at 4°C; (3) PBS, 6 × 5 min each; (4) appropriate secondary antibodies in blocking solution for 6-8 hr at room temperature; (5) PBS, 6 × 5 min each.

**Figure 9:**
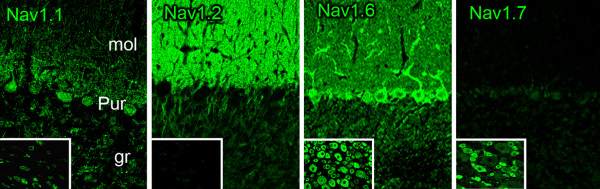
**Sodium channel immunolabeling in rat cerebellum and DRG**. Isoform-specific antibodies generated against sodium channels Na_v_1.1, Na_v_1.2, Na_v_1.6 and Na_v_1.7 were reacted with sections of adult rat cerebellum and DRG. Purkinje cell bodies and apical dendrites exhibit robust Na_v_1.1 immunolabeling, while limited Na_v_1.1 immunoreactivity is displayed in DRG neurons (inset). Parallel fibers of cerebellar granule cells exhibit substantial Na_v_1.2 labeling; Na_v_1.2 is not detectable in DRG neurons (inset). Na_v_1.6 is robustly expressed in Purkinje cell bodies and dendrites and is also localized within parallel fibers of cerebellar granule cells; Na_v_1.6 immunolabeling is exhibited by most neurons within DRG (inset). Within cerebellum, Na_v_1.7 immunostaining is not detectable, but Na_v_1.7 immunoreactivity is exhibited by many DRG neurons. The labeling patterns obtained with the isoform-specific sodium channel antibodies Na_v_1.1, Na_v_1.2, Na_v_1.6 and Na_v_1.7 utilized in these studies is consistent with previous descriptions of their localization within CNS and PNS tissue [4,29].

Sections of rat DRG and cerebellum were also reacted with the antibodies to serve as specificity controls. Figure 9 shows immunostaining pattern that is consistent with the known distribution of these channels in DRG and cerebellum [4,29]. We have previously shown that rabbit anti-Na_v_1.6 (1:100, Sigma) does not stain nodes of Ranvier from *Scn8a*^*medtg *^mice [61] which lack Na_v_1.6 channels [62]. Additional control experiments were performed without inclusion of primary antibodies, which yielded only background levels of fluorescence (data not shown). Tissue sections were examined with a Nikon C1 confocal microscope (Nikon USA, Melville, NY).

### Voltage-clamp recordings from OSN cultured from adult mice

OSN cultures from adult C57BL/6 mice were done according to report by Sosnowski et al [63] with some modifications. In brief, mice were anesthetized with ketamine/xylazine (100/10 mg/kg, i.p.), decapitated, and olfactory tissue was dissected and immediately placed in ice-cold Ca^2+^/Mg^2+^-free HBSS. After freeing olfactory epithelium from other tissues, olfactory epithelium was rinsed in ice-cold Ca^2+^/Mg^2+^-free HBSS and minced to 1 mm pieces. Trypsin (0.125% in Ca^2+^/Mg^2+^-free HBSS) treatment was used to dissociate epithelial tissue at 35°C for 30 min with gentle agitation. Trypsin was inactivated by OSN medium (MEM containing 10% fetal bovine serum and antibiotics). Dispersed cells were mixed gently and centrifuged at 1200 × g for 2 min. The pellet was resuspended in 1 ml OSN medium with a fire-polished glass pipette and filtered through a 40-μm mesh. Approximately 50 μl of cell suspension was plated on a poly-D-lysine/laminin coated coverslip in 24-well plate (BD Biosciences). Cultures were placed in a humidified 35°C incubator receiving 5% CO_2_. One hour later, cells were fed with 450 μl of OSN medium with fresh NGF (50 ng/ml). Half the medium was replaced daily and supplied with NGF. Cultures were used for patch-clamp recording on the same day of culture.

The whole-cell voltage-clamp recording was conducted at room temperature (21-23°C) using an Axopatch 200B amplifier (Molecular Devices, Union City, CA). The bath solution contained (in mM): 140 NaCl, 3 KCl, 1 CaCl_2_, 1 MgCl_2_, 10 glucose and 20 HEPES, pH 7.3 with NaOH (adjusted to 320 mOsm with sucrose), and the pipette solution contained (in mM): 140 CsF, 1 EGTA, 10 NaCl, 10 mM HEPES, pH 7.3 with CsOH (adjusted to 310 mOsm with sucrose). 20 mM TEA-Cl was included in the bath solution to block endogenous potassium current. Fire-polished electrodes were fabricated from capillary glass (PG10165-4, World Precision Instruments, Sarasota, FL) using a P-97 puller (Sutter Instrument Co., Novato, CA). The resistance of recording pipettes in the bath solution was 2-5 MΩ. Whole-cell capacitive currents were compensated with analog compensation and 60-80% series resistance compensation was applied to minimize voltage errors. The voltages were not corrected for liquid junction potential. The currents were filtered at 5 kHz, acquired at 100 kHz, and then digitized using pClamp 10 software and Digidata 1440A (Molecular Devices). The Origin 8.1 software (OriginLab Corporation, Northampton, MA) was used for data analysis. Data are presented as means ± S.E.

## Abbreviations

IEM: inherited erythromelalgia; PEPD: paroxysmal extreme pain disorder; CIP: congenital insensitivity to pain; OSN: olfactory sensory neuron; OMP: olfactory mature protein; ONL: olfactory nerve layer; MAP2: microtubule associated protein 2; DRG: dorsal root ganglion; SCG: superior cervical ganglion; TTX: tetrodotoxin; TTX-S: tetrodotoxin-sensitive; TTX-R: tetrodotoxin-resistant.

## Competing interests

The authors declare that they have no competing interests.

## Authors' contributions

HA acquired and analyzed electrophysiology data, established OSN cell culture, and participated in writing the manuscript. JAB designed immunocytochemical experiments, acquired and analyzed and interpreted data, and participated in writing the manuscript. PZ established OSN cultures, acquired and analyzed multiplex RT-PCR and immunohistochemical data. LT designed, acquired and analyzed molecular data to determine the identity of sodium channels in OSN and DRG. SCG and SDH conceived and coordinated the study and wrote and edited the manuscript. All authors read and approved the final manuscript.
